# Decline of *Salmonella enterica* Serotype Choleraesuis Infections, Taiwan

**DOI:** 10.3201/eid2004.130240

**Published:** 2014-04

**Authors:** Lin-Hui Su, Tsu-Lan Wu, Cheng-Hsun Chiu

**Affiliations:** Chang Gung Memorial Hospital, Chang Gung University College of Medicine, Taoyuan, Taiwan (L.-H. Su, T.-L. Wu);; Chang Gung Children’s Hospital, Chang Gung University College of Medicine, Taoyuan (C.-H. Chiu)

**Keywords:** Salmonella enterica serotype Choleraesuis, invasive infection, antimicrobial resistance, bacteria, Taiwan, zoonoses

**To the Editor:** Human salmonellosis is a major public health problem of global concern. *Salmonella* *enterica* serotype Choleraesuis is a nontyphoid serotype; it has a narrow host range and is associated with a high proportion of invasive infections (50%–90% of isolates are from sterile sites [(*1*)]). In 2000, emergence of fluoroquinolone resistance in *S. enterica* Choleraesuis was reported from Chang Gung Memorial Hospital at Linkou, Taiwan (*2*). Since then, we have continuously monitored the trend of these infections at this hospital in northern Taiwan and at Chang Gung Memorial Hospital at Kaohsiung in southern Taiwan. We report the decline of *S. enterica* Choleraesuis infections coinciding with the implementation of several national control programs in Taiwan.

In humans, *S. enterica* Choleraesuis usually causes invasive infections (*1*). Transmission of the organism is probably from pigs to humans (*3*). According to an inspection program conducted by the Council of Agriculture in 2004, illegal slaughter of pigs dying of unidentified diseases and sale of the pork at a reduced price were common in Taiwan and probably were associated with transmission of the organism (*4*). In Taiwan during 1996–1997, a large epidemic of swine foot-and-mouth disease occurred (*5*), and consumption of pork decreased substantially. To prevent further spread of that epidemic, the government implemented massive slaughter of infected pigs and a swine vaccination policy (*5*). Coincidently, in 1996–1998, *S. enterica* Choleraesuis infections among humans decreased significantly (p<0.001) (*2*). However, during the following years, infections among humans and antimicrobial drug resistance gradually increased (*2*). In Taiwan, widespread use of antimicrobial drugs in animals, which might contribute to development of antimicrobial drug resistance in human pathogens, was subsequently reported (*6*).

The Taiwan government took further actions to improve the quality of pig husbandry. In 2002, a live attenuated *S. enterica* Choleraesuis vaccine (Suisaloral; Impfstoffwerk Dessau-Tornau GmbH, Rosslay, Germany) for pigs was licensed for importation (veterinary drug license no. 06387) (*7*). In 2004, the following approaches were implemented to consolidate the management of animal husbandry and ensure the quality and safety of meat: improving pollution control and sanitary conditions in pig farms by periodic inspection and education programs; implementing regulations to promote and improve an existing quality food certification system, Certified Agricultural Standards, by tracking and auditing illicit labeling; reinforcing monitoring and tracking systems for the sale and consumption of antimicrobial drugs for animal use (by continuing education and inspections with or without fixed schedules); preventing illegal slaughtering and sale of pigs dying of unidentified diseases (by inspections and monetary penalties for violations); and establishing a Taiwan Agriculture and Food Traceability system (http://taft.coa.gov.tw) to fully record all processes from farm to table (*4*,*8*). A particularly notable control measure launched at that time was a death insurance program for pigs. Although farmers were fully compensated for their losses, pigs dying of unidentified diseases were collected and chemically marked to prevent the possibility of subsequent illegal use (*4*). In 2005, 2 representative counties in central-southern Taiwan tried the program. During the next year, the program was extended to 8 neighboring counties, and in 2007, it was implemented in all counties (*9*). Furthermore, since the establishment of the Taiwan Agriculture and Food Traceability system, various Taiwan Good Agricultural Practice manuals have been gradually developed, and in 2008, a manual for pig farming established official standard operating procedures (*10*).

The control measures seem to be successful, as evidenced by the results of a long-term monitoring program at the 2 large tertiary care hospitals in northern and southern Taiwan (Figure). *S. enterica* Choleraesuis infections have declined significantly since 2005 in southern Taiwan (Figure, panel A) and since 2006 in northern Taiwan (Figure, panel B) (χ^2^ test for trend, p<0.01). At the southern hospital, the number of nonrepetitive clinical isolates was reduced by almost half in 2005 (Figure, panel A), the year the death insurance program was initiated in central-southern Taiwan. The reduction was even larger at the other hospital. The annual number of clinical isolates obtained decreased from >80 in 2004–2005 to 24 in 2006 (Figure, panel B), when the death insurance program was expanded to more areas. At both hospitals, the annual number of *S. enterica* Choleraesuis infections had decreased consistently in the subsequent years; since 2008, this number has remained <10 (Figure).

**Figure:**
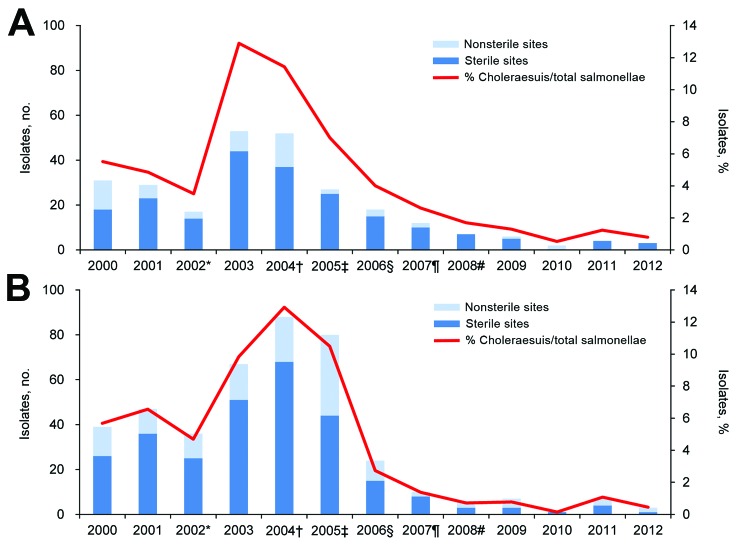
Trends of annual numbers and percentages of *Salmonella enterica* serotype Choleraesuis isolates from 2 tertiary care hospitals in Taiwan. A) Data from Chang Gung Memorial Hospital at Kaohsiung, southern Taiwan. B) Data from Chang Gung Memorial Hospital at Linkou, northern Taiwan. *Approval and importation of vaccine for swine. †Promotion of the Certified Agricultural Standards quality food certification system (*4*), monitoring of sale of antimicrobial drugs for animal use (*4*), inspection of chemical residues in swine farms and pork market, launch of educational programs about safe use of drugs in animals (*4*); inspection of illegal slaughtering and sale of farmed animals dying of unidentified disease (*4*); and establishment of Taiwan Agriculture and Food Traceability system (*4**,**8*). ‡Initiation of death insurance program for pigs in 2 representative central-southern counties (*9*). §Extension of death insurance program to another 8 neighboring counties (*9*). ¶Full implementation of death insurance program throughout all Taiwan counties (*9*). #Establishment of Taiwan Good Agricultural Practice for pig husbandry (*10**).*

After interruption of the identified infection chain, the substantial decline of infection among humans became evident. Despite the absence of national surveillance data for nontyphoid human salmonellosis in Taiwan, the decrease in *S. enterica* Choleraesuis infections reported herein has also been noted at other hospitals in Taiwan (L.-H. Su, pers. comm.). This finding demonstrates that application of effective control measures on farms and in agricultural practices can lead to successful control of *S. enterica* Choleraesuis infection among humans.
